# Coronal mass ejections are not coherent magnetohydrodynamic structures

**DOI:** 10.1038/s41598-017-04546-3

**Published:** 2017-06-23

**Authors:** M. J. Owens, M. Lockwood, L. A. Barnard

**Affiliations:** Space and Atmospheric Electricity Group, Department of Meteorology, University of Reading, Earley Gate, PO Box 243, Reading, RG6 6BB UK

## Abstract

Coronal mass ejections (CMEs) are episodic eruptions of solar plasma and magnetic flux that travel out through the solar system, driving extreme space weather. Interpretation of CME observations and their interaction with the solar wind typically assumes CMEs are coherent, almost solid-like objects. We show that supersonic radial propagation of CMEs away from the Sun results in geometric expansion of CME plasma parcels at a speed faster than the local wave speed. Thus information cannot propagate across the CME. Comparing our results with observed properties of over 400 CMEs, we show that CMEs cease to be coherent magnetohydrodynamic structures within 0.3 AU of the Sun. This suggests Earth-directed CMEs are less like billiard balls and more like dust clouds, with apparent coherence only due to similar initial conditions and quasi homogeneity of the medium through which they travel. The incoherence of CMEs suggests interpretation of CME observations requires accurate reconstruction of the ambient solar wind with which they interact, and that simple assumptions about the shape of the CMEs are likely to be invalid when significant spatial/temporal gradients in ambient solar wind conditions are present.

## Introduction

Coronal mass ejections (CMEs) are large, episodic eruptions of coronal plasma and magnetic flux that are ejected out into the heliosphere at speeds typically^1^ ranging from 300–2000 km s^−1^. They are of great interest both for their central role in extreme space weather^2, 3^ and in the solar cycle evolution of the coronal magnetic field^4, 5^. *In situ* spacecraft observations of CMEs show that around a third to a half of all CMEs contain a magnetic flux-rope structure and low plasma beta^6, 7^. These “magnetic clouds” are generally assumed to be (quasi-) coherent magnetohydrodynamic (MHD) structures, wherein the magnetic pressure and curvature forces act, to a greater or lesser extent, to resist deformation by external forces such as solar wind speed shear. This, in principle, enables a magnetic cloud to evolve as a single cohesive body. For example:

- Observations of CME-CME interactions in the heliosphere^8^ have been interpreted as elastic or even super-elastic collisions^9^, suggesting the CMEs are solid-like, coherent structures.
- Non-radial deflection of CME trajectories, possibly by interaction with coronal hole magnetic flux, has been observed^10–12^. While this has largely been interpreted as centre-of-mass deflection, which would require the CME to behave as a coherent structure, distortion of the CME shape could equally explain the available observations.
- Methods for tracking CMEs through the corona and heliosphere assume the CME front remains quasi-spherical (or some other simple shape)^13–16^, implying the CME front remains a coherent structure throughout the heliosphere. There is observational evidence, however, for significant disruption of CME structure by solar wind inhomogeneity^17^.
- Numerous studies (including some by the authors of present paper) either explicitly or implicitly assume that single-point *in situ* measurements of a magnetic cloud are representative of its global structure^7, 18–24^, implying a large degree of coherence of CMEs. Single-^25^ and multi-point^26, 27^ observations, even at relatively modest spacecraft separations, often reveal this picture to be far too simplistic, with evidence of CME distortion by the ambient solar wind.

Numerical MHD models provide a complementary means to test the coherence of CMEs. There have been a number of numerical experiments investigating interaction of CMEs both with a structured solar wind and other CMEs, which often reveal significant distortion of CME structure^28–33^. Interpretation of the results, however, has largely focussed on the issue of force balance, with internal magnetic pressure/curvature from the magnetic flux-rope unable to resist distortion from interaction with external solar wind structures.

Here, we investigate a fundamental physical limit on a CME’s ability to act as a coherent magnetohydrodynamic structure; namely the inability of information to propagate within a CME. We use a simple analytical model for CME evolution in the heliosphere to calculate the Alfvén wave speed [*V*_*A*_] within the CME at a range of heliocentric distances. We also estimate the geometric speed of separation of plasma parcels [*V*_*G*_] within the CME that results from purely radial heliocentric propagation. For a range of CME parameters, we determine the heliocentric distance at which *V*_*G*_ exceeds *V*_*A*_ and hence information can no longer propagate within the CME.

## Methodology

**Figure 1:**
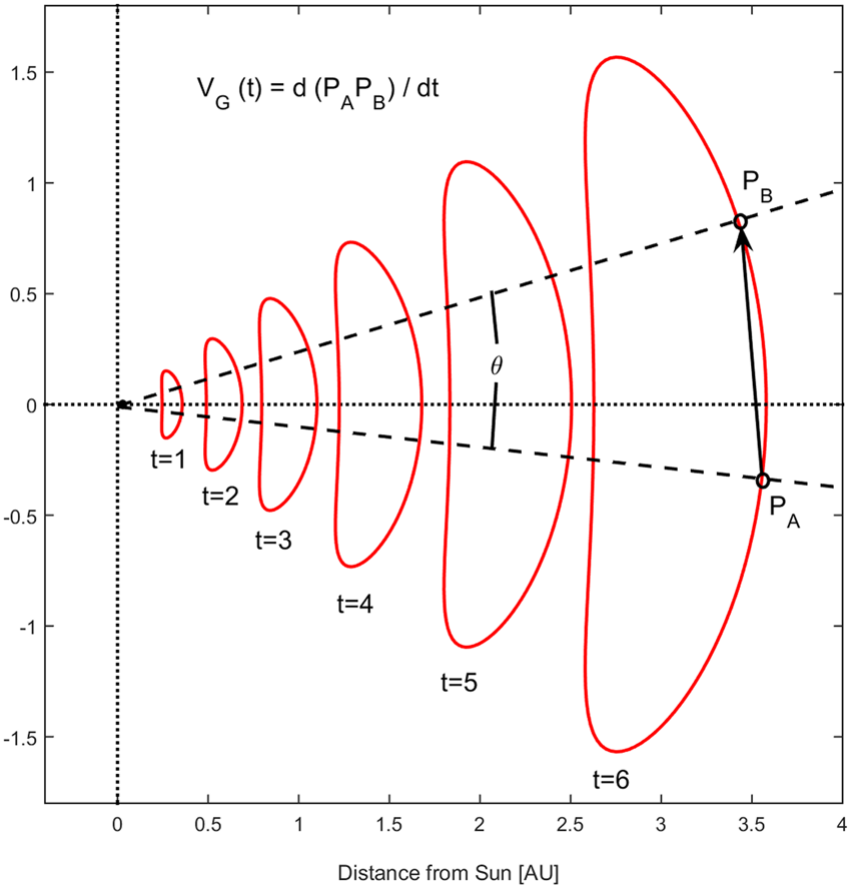
An analytical model for the cross-sectional area of a CME as it propagates anti-sunward. Snapshots are shown at successive times. The plane is perpendicular direction of propagation (e.g., the ecliptic or RN planes in heliocentric radial-tangential-normal, RTN, coordinates). Points *P*_*A*_ and *P*_*B*_ on the leading edge of the CME subtend an angle *θ* at the centre of the Sun. Due to radial propagation in spherical geometry, *P*_*A*_ and *P*_*B*_ separate with time, leading to the geometric speed *V*_*G*_.

**Figure 2:**
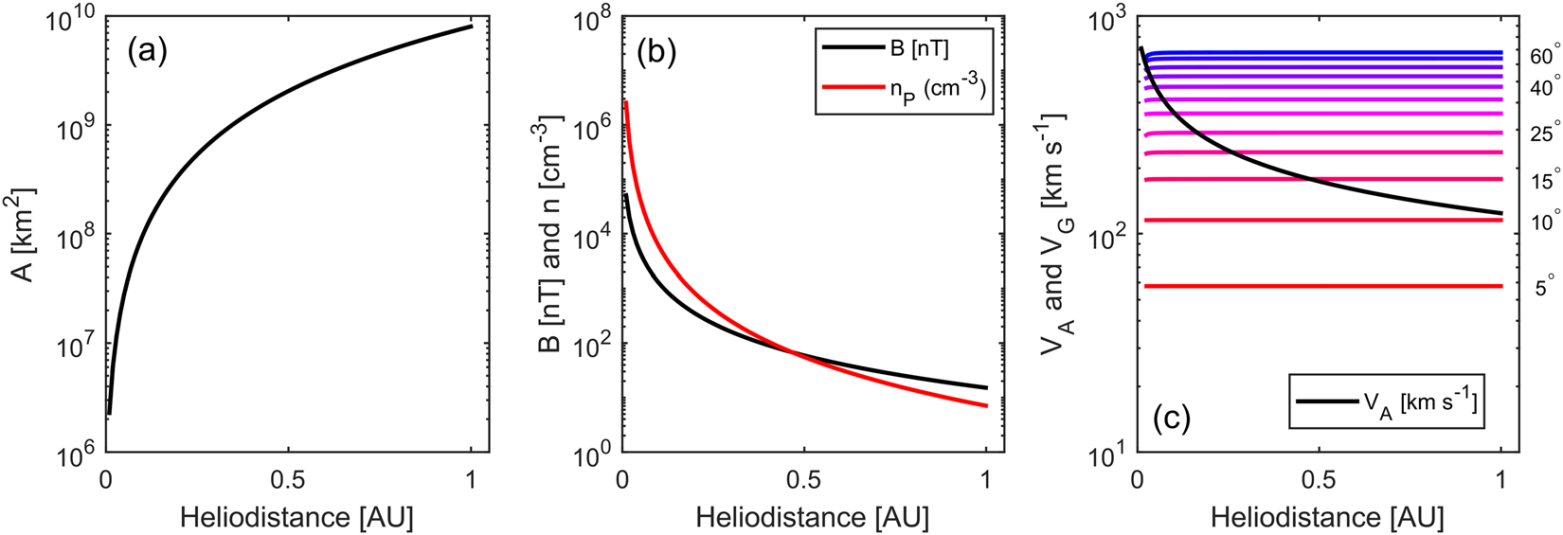
Evolution of CME properties with heliocentric distance, using *V*_*TR*_ = 600 km s^−1^, *B*_*1AU*_ = 15 nT and *n*_*1AU*_ = 7 cm^−3^. Panel (a) shows the cross-sectional area of the CME. Panel (b) shows the magnetic field intensity (*B*, in black), assuming constant magnetic flux threading the CME cross section, and the ion number density (*n*, in red), assuming conservation of mass within the CME. Panel (c) shows the resulting Alfven speed within the CME (*V*_*A*_, black). Coloured lines show the geometric separation speeds [*V*_*G*_] of points on the CME leading edge as a result of expansion in spherical geometry for a range of separation angles [*θ*], from 5° (red) to 60° (blue) in 5° steps.

The geometric and dynamic effects of CME propagation are investigated using a simple analytical model, closely following Owens, *et al*.^21^, which agrees well with numerical MHD simulations of CME evolution^34^. In summary, CMEs are assumed to initially take the form of a circular cross-section, force-free flux rope in the low corona and subsequently be deformed by a combination of CME-centred self-expansion and heliocentric radial propagation. The internally-driven self-expansion is limited to the heliocentric radial direction, so that the CME maintains constant angular width, as is commonly observed^1^. Figure 1 shows snapshots of the resulting CME cross section at increasing times (in arbitrary units), using typical CME parameters: an initial (at time t = 0) circular cross-section of radius 1 solar radii [*r*_*S*_] at a height of 2 *r*_*S*_ gives a CME angular extent with respect to the Sun of approximately 60°; a constant CME transit speed [*V*_*TR*_] of 600 km s^−1^ and a constant internally-driven expansion speed [*V*_*EX*_] of 90 km s^−1^ ^35^. The CME rapidly “pancakes” due to radial propagation in spherical geometry^34, 36^. The change in CME cross-sectional area, computed by numerically integrating the analytical model, is shown in Fig. 2a. By 1 AU, the cross-sectional area of the CME is approximately 3000 times its initial value.

From this model and a number of reasonable assumptions, it is possible to estimate the bulk properties within the evolving CME and so compute the Alfvén speed. We assume that the total magnetic flux within the CME is conserved (true to within a few percent^37^) and that the magnetic flux is orientated perpendicular to the CME cross section. Thus *B*, the magnetic field intensity within the CME at a heliocentric distance *R*, will scale with the CME cross-sectional area, *A*:

1 $$B={B}_{0}\frac{{A}_{0}}{A}$$

where the subscript *0* refers to values at a reference distance. Figure 1b shows the profile for *B*_*0*_ = 15 nT at *R*_*0*_ = 1 AU, a typical value observed *in situ* ^35^.

Similarly, if the amount of plasma within the CME is assumed to be constant, the ion density [*n*] at distance *R* will scale as the volumetric increase of the CME:

2 $$n={n}_{0}\frac{{A}_{0}{R}_{0}}{A\,R}$$

The black line in Fig. 2b shows the *n* profile for a CME proton density of *n*_*0*_ = 7 cm^−3^ at *R*_*0*_ = 1 AU, again a typical observed value^38^. Combining these two parameters allows approximation of the Alfvén speed [*V*_*A*_] within a CME as a function of heliocentric distance, *R*:

3 $${V}_{A}=\frac{B}{\sqrt{{\mu }_{0}n\,{m}_{i}}}$$

where *μ*_*0*_ is the magnetic permeability of free space and *m*_*i*_ is the mean ion mass. For simplicity we here assume a proton ion plasma which gives an upper limit for the Alfvén speed: for helium ion composition of 8%, m_i_ is 1.24 a.m.u. and the Alfvén speed would be 0.9 times the values given here. Note that the maximum wave speed within a magnetised plasma is the fast magnetosonic speed, a combination of *V*_*A*_ and the ion-acoustic wave speed [*V*_*S*_] which results from the finite plasma temperature. Using a typical 1-AU temperature and a polytropic index as high as 4/3, *V*_*S*_ within a CME remains at least an order of magnitude lower than *V*_*A*_ at all heliocentric distances, so can be ignored for the purposes required here.

## Results

The black line in Fig. 2c shows *V*_*A*_ as a function of heliocentric distance. The coloured lines show the separation speed [*V*_*G*_] of points on the CME leading edge which results from radial expansion in spherical geometry. The red line shows points separated by a heliocentric angle *θ* = 5°, while the blue line shows *θ* = 60°, the angular extent of a typical CME^1^. Coloured lines show separations in 5° steps between these two limits.

For small values of *θ* (<10°), the Alfven speed is greater than the geometric separation speed for the entirety of the CME’s transit to 1 AU. For plasma parcels separated by *θ* = 15°, a quarter of the total angular extent of a typical CME, *V*_*G*_ first exceeds *V*_*A*_ at approximately 0.45 AU. We refer to this distance as the critical distance [*R*_*CRIT*_] as once this *V*_*G*_ > *V*_*A*_ conidtion is met information can no longer travel between plasma parcels of the given angular separation and the CME has lost coherence over such length scales. For increasing angular separations, this critical distance moves ever closer to the Sun. For *θ* = 60°, the typical CME angular width, magnetic coherence is lost almost immediately after eruption, at least in this example (i.e., for CME transit speed of 600 km s^−1^ and *B* at 1 AU of 15 nT).

**Figure 3:**
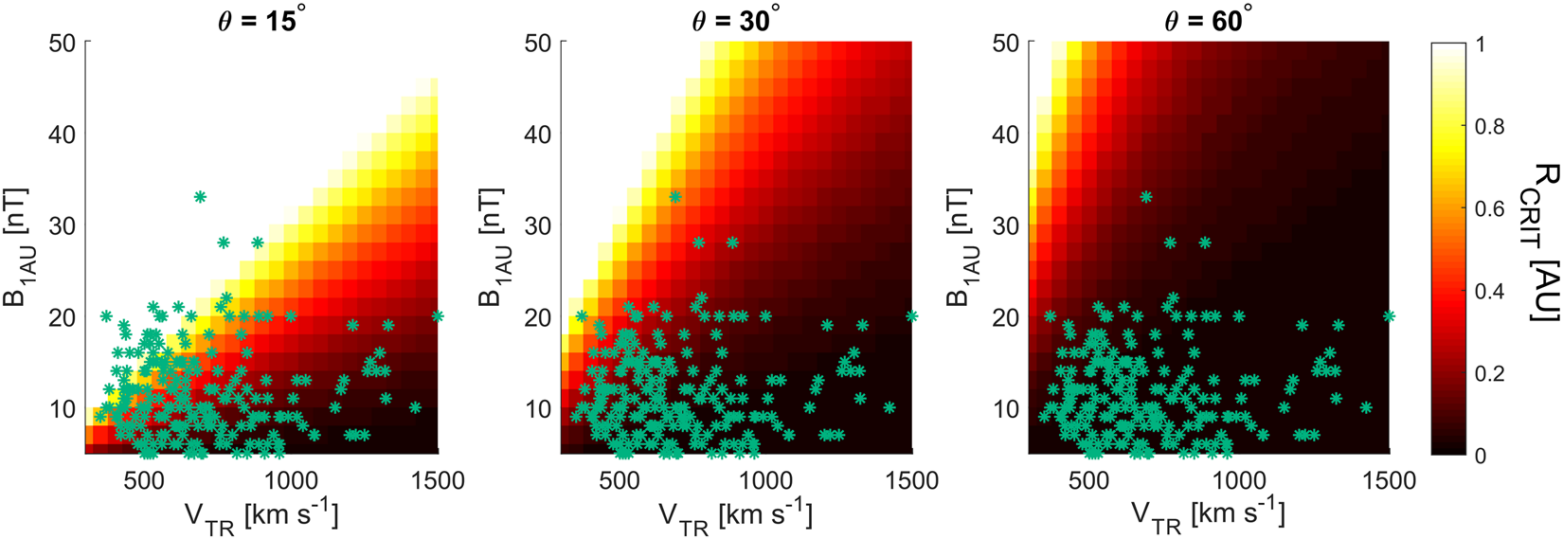
The critical distance, *R*_*CRIT*_, at which expansion speed exceeds the Alfven speed and the CME ceases to be a coherent structure, as a function of CME transit speed [*V*_*TR*_] and the magnetic field intensity within a CME at 1 AU [*B*_*1AU*_]. The panels, from left to right, show angular separations on the CME front of 15°, 30° and 60°, respectively. These correspond to a quarter, half and the full angular extent of a typical CME, respectively. The cyan dots show CME observations from the Cane and Richardson^38^ catalogue, updated to the end of 2016.

We now investigate the effect of CME properties on the critical distance. Figure 3 shows *R*_*CRIT*_ as a function of CME transit speed [*V*_*TR*_] and magnetic field intensity at 1 AU [*B*_*1AU*_]. *n* is fixed at 7 cm^−3^, though similar results are found for a reasonable range of *n*. Panels, from left to right, show angular separations of 15°, 30° and 60°. These correspond to a quarter, half and the full angular extent of a typical CME, respectively. The general trend is for *R*_*CRIT*_ to increase with CME magnetic field intensity and to decrease with CME transit speed. For extremely narrow CMEs (~15°), or plasma parcels within a typical CME that are separated by approximately a quarter of the total angular extent, *V*_*A*_ can remain above *V*_*G*_ out to 1 AU as long as the CME speed is relatively low and the magnetic field intensity is relatively high. The blue dots in Fig. 3 show values of *B*_*1AU*_ and *V*_*TR*_ from observations of 477 CMEs, obtained by combining coronagraph and *in situ* over the period 1995–2016^38^. Only a small fraction of these observed CMEs (<10%) have properties which suggest they remain coherent over an angular extent of 15° out to 1 AU. The bulk of the CMEs, approximately 70%, have lost coherency across 15° of angular extent within 0.4 AU. Increasing the angular separation to 30°, about half the angular extent of a typical CME, none of the observed CMEs remain coherent to 1 AU, with most losing coherence within 0.2 AU. Finally, looking at the full angular extent of a typical CME, 60°, all observed CMEs have lost coherence by 0.3 AU, with ~90% losing coherence within 0.1 AU.

## Discussion and Conclusions

**Figure 4:**
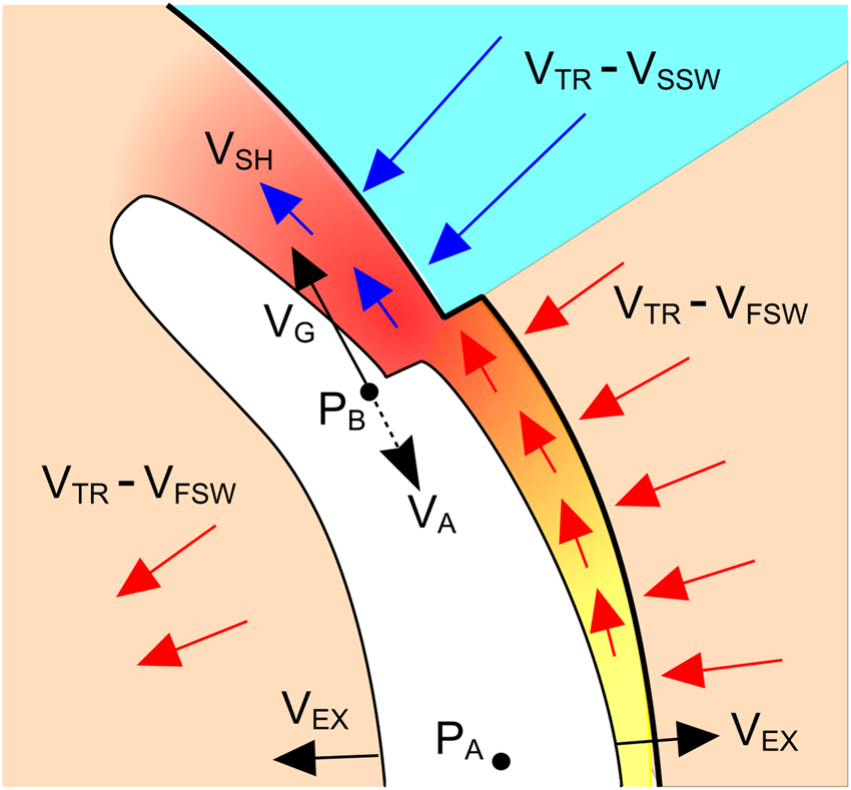
A schematic of one flank of a CME (white) propagating through a structured solar wind, in the reference frame of a point *P*_*A*_, located close to the centre of the CME. The shock (thick black line), and CME leading/trailing edges move away from *P*_*A*_ at the CME expansion speed, *V*_*EX*_. Fast solar wind, in beige, flows into the CME shock at a speed *V*_*TR*_ + *V*_*EX*_ − *V*_*FSW*_ (*V*_*TR*_ and *V*_*FSW*_ are the CME transit speed and the fast solar wind speed, respectively). Slow solar wind, in blue, flows into the shock at a speed of *V*_*TR*_ + *V*_*EX*_ − *V*_*SSW*_, (where *V*_*SSW*_ is the slow solar wind speed). The point *P*_*B*_, located at the fast/slow solar wind interface, experiences a distortion of the CME magnetic field and launches an Alfven wave at speed *V*_*A*_ towards *P*_*A*_. Point *P*_*B*_, however, is moving away from *P*_*A*_ due to geometric expansion at a speed *V*_*G*_, thus the information can never arrive. Similarly, *V*_*SH*_, the speed of the deflected solar wind flow in the sheath behind the shock, is smaller than *V*_*G*_ and thus the sheath flow cannot travel around the CME.

This study has investigated the speed at which information can propagate between CME plasma parcels (the Alfvén speed, *V*_*A*_), relative to the speed at which CME plasma parcels separate owing to radial propagation in spherical geometry [*V*_*G*_]. Where *V*_*G*_ exceeds *V*_*A*_, plasma parcels can no longer be considered to constitute a single, coherent magnetohydrodynamic (MHD) structure. Figure 4 illustrates this idea. It shows a CME travelling through fast solar wind, but the upper flank encounters a slow wind stream. This results in distortion of the magnetic field structure within the CME. An Alfven wave is launched at a speed *V*_*A*_ from point *P*_*B*_, which lies within the CME at the latitude of the solar wind speed shear, towards a point *P*_*A*_, located near the centre of the CME. Geometric expansions means that *P*_*B*_ is moving away from *P*_*A*_ at a speed *V*_*G*_. If *V*_*G*_ > *V*_*A*_, as shown in this example, information cannot travel between the two points. Thus *P*_*A*_ and *P*_*B*_ are effectively isolated, and the response of the CME at points *P*_*A*_ and *P*_*B*_ to a structured solar wind is entirely independent; there can be no action as a single body, regardless of the magnitude of restoring forces such as magnetic pressure and curvature forces. A similar effect is expected within the deflected solar wind flow in the sheath region ahead of a fast moving CME^39^. Due to the large *V*_*G*_, the deflected solar wind flow within the sheath (labelled *V*_*SH*_ in Fig. 4)^24^ cannot keep pace with a point on the leading edge and thus does not flow around the obstacle, but piles up ahead of it.

We estimate *V*_*A*_ and *V*_*G*_ using an analytic model, allowing parameter space to be fully and efficiently explored. Where simplifying assumptions are required, they have been chosen as far as possible to act in the favour of CME coherence (e.g., limiting the expansion of CMEs to the radial direction reduces V_G_; coherence is defined to be lost when V_G_ exceeds V_A_, rather than when the information travel time becomes large compared to the CME life time; helium is not included in the Alfvén speed estimation, etc). Thus we effectively examine the “best case scenario” for CME coherence. Nevertheless, we find that all observed CMEs lose coherence over their full angular extent by 0.1 to 0.2 AU. Even considering Alfvén wave propagation over half the typical CME angular extent, which would allow, e.g., the east flank of an ICME to know what’s happening to the west flank, no observed CMEs are expected to maintain coherence to 1 AU; indeed, less than 0.5% of all observed CMEs are expected to maintain flank-to-flank coherence past 0.3 AU.

One aspect that requires further investigation is the assumption that the fastest information path between two points is a straight line. While this is true for the analytical model employed here, as it has constant magnetic field intensity within a CME, in a real magnetic cloud this need not be the case. For an ideal force-free magnetic flux rope, the magnetic field intensity is highest at the flux rope axis (i.e., the centre of the CME). Thus shorter information travel times between two points on the CME leading edge could, in principle, be obtained using a non-linear ray path taking advantage of the increased Alfvén speed deep within the CME. An alternative preferential wave path could be through the high magnetic field intensities in the sheath region ahead of a fast CME, though the sheath is often high plasma density too, meaning the Alfvén speed may not be enhanced. These dynamic effects will be fully investigated using numerical magnetohydrodynamic modelling of an erupting magnetic flux rope and ray-tracing at each time step. In practice, however, these effects are unlikely to provide significantly different results to those presented here. Any increased Alfvén speed will be offset by an increased path length, and compression of the CME leading edge by interaction with the ambient solar wind means the highest magnetic field intensities are usually located near the CME leading edge, not near the centre of the CME^35^.

In light of these findings, new approaches are required for the interpretation of CME observations. We discuss a few examples here. The highly structured intensity patterns routinely seen within CMEs in Heliospheric Imager (HI) observations^40^ by the STEREO spacecraft may be a direct result of both the scale of coherence within a CME and the variability of the solar wind through which a CME is travelling. These relatively small-amplitude, small-scale structures are unlikely to be a significant issue for interpretation of the global properties of CMEs, either with the geometric models applied to HI observations to determine CME speed and direction^13^, or to flux-rope models applied to *in situ* observations^18^. Larger amplitude gradients in the solar wind, however, such as a sharp latitudinal or longitudinal transition between fast and slow wind (Fig. 4), are likely to invalidate both forms of reconstruction technique by generating both large distortion to the CME shape and radically altering the pile-up of the solar wind plasma in the CME sheath, which is the plasma that is imaged by Thompson-scattered photospheric light. The results presented here also suggest CME arrival-time forecasting is sensitive to ambient solar wind structure at the local scale, not just at a global scale^41^: application of a drag equation to a CME’s interaction with the solar wind^42^ is only really valid along an individual radial flow line, not to the CME as a whole. We suggest CME reconstruction techniques need to be modified to incorporate information about solar wind structure, either from global MHD models or from previous solar wind observations (e.g., assuming corotation of the solar wind). Ultimately, this may require solar wind data assimilation, to best interpolate and extrapolate between the available observations using physics-based models^32^.

## References

[1] Yashiro, S. *et al*. A catalog of white light coronal mass ejections observed by the SOHO spacecraft. *J. Geophys. Res*. **109**, doi:10.1029/2003JA010282 (2004).

[2] Gosling JT (1993). The solar flare myth. J. Geophys. Res..

[3] Cannon, P. *et al*. *Extreme space weather: impacts on engineered systems and infrastructure* (Royal Academy of Engineering, 2013).

[4] Low BC (2001). Coronal mass ejections, magnetic flux ropes, and solar magnetism. J. Geophys. Res..

[5] Owens MJ, Crooker NU (2006). Coronal mass ejections and magnetic flux buildup in the heliosphere. J. Geophys. Res..

[6] Klein LW, Burlaga LF (1982). Interplanetary magnetic clouds at 1 AU. J. Geophys. Res..

[7] Burlaga LF (1988). Magnetic clouds: Constant alpha force-free configurations. J. Geophys. Res..

[8] Lugaz N (2012). The deflection of the two interacting coronal mass ejections of 2010 May 23-24 as revealed by combined *in situ* measurements and heliospheric imaging. Astrophys. J..

[9] Shen C (2012). Super-elastic collision of large-scale magnetized plasmoids in the heliosphere. Nature Physics.

[10] Kay, C., Opher, M. & Evans, R. M. Global Trends of CME Deflections Based on CME and Solar Parameters. *The Astrophysical Journal* **805**, doi:10.1088/0004-637X/805/2/168 (2015).

[11] Byrne, J. P., Maloney, S. A., McAteer, R. T. J., Refojo, J. M. & Gallagher, P. T. Propagation of an Earth-directed coronal mass ejection in three dimensions. *Nature Communications* **1**, doi:10.1038/ncomms1077 (2010). 10.1038/ncomms1077 20865805

[12] Möstl, C. *et al*. Strong coronal channelling and interplanetary evolution of a solar storm up to Earth and Mars. *Nature Communications* **6**, doi:10.1038/ncomms8135 (2015). 10.1038/ncomms8135 PMC4455070 26011032

[13] Möstl C, Davies JA (2013). Speeds and Arrival Times of Solar Transients Approximated by Self-similar Expanding Circular Fronts. Solar Physics.

[14] Davies, J. A. *et al*. A Self-similar Expansion Model for Use in Solar Wind Transient Propagation Studies. *The Astrophysical Journal* **750**, doi:10.1088/0004-637X/750/1/23 (2012).

[15] Liu Y (2010). Reconstructing Coronal Mass Ejections with Coordinated Imaging and *in Situ* Observations: Global Structure, Kinematics, and Implications for Space Weather Forecasting. The Astrophysical Journal.

[16] Thernisien A, Vourlidas A, Howard RA (2009). Forward Modeling of Coronal Mass Ejections Using STEREO/SECCHI Data. Solar Physics.

[17] Savani NP, Owens MJ, Rouillard AP, Forsyth RJ, Davies JA (2010). Observational Evidence of a Coronal Mass Ejection Distortion Directly Attributable to a Structured Solar Wind. Astrophys. J. Lett..

[18] Lepping RP, Jones JA, Burlaga LF (1990). Magnetic field structure of interplanetary clouds at 1 AU. J. Geophys. Res..

[19] Farrugia, C. J., Osherovich, V. A. & Burlaga, L. F. Magnetic flux rope versus the spheromak as models for interplanetary magnetic clouds. *J. Geophys. Res*. **100**, 12293-+, doi:10.1029/95JA00272 (1995).

[20] Démoulin P, Dasso S (2009). Magnetic cloud models with bent and oblate cross-section boundaries. Astron. & Astrophys.

[21] Owens MJ, Merkin VG, Riley P (2006). A kinematically distorted flux rope model for magnetic clouds. J. Geophys. Res..

[22] Hu Q, Sonnerup BUO (2001). Reconstruction of magnetic flux ropes in the solar wind. Geophys. Res. Lett..

[23] Hidalgo MA, Cid C, Vinas AF, Sequeiros J (2002). A non-force-free apporach to the topology of magnetic clouds. J. Geophys. Res..

[24] Owens MJ, Cargill PJ (2004). Non-radial solar wind flows induced by the motion of interplanetary coronal mass ejections. Ann. Geophys..

[25] Owens, M. J. Magnetic cloud distortion resulting from propagation through a structured solar wind: Models and observations. *J. Geophys. Res*. **111**, doi:10.1029/2006JA011903 (2006).

[26] Mulligan T, Russell CT (2001). Mulitspacecraft modeling of the flux rope structure of interplanetary coronal mass ejections: Cylindrical symmetric versus nonsymmetric topologies. J. Geophys. Res..

[27] Kilpua E (2009). Multispacecraft observations of magnetic clouds and their solar origins between 19 and 23 May 2007. Solar Physics.

[28] Schmidt JM, Cargill PJ (2001). Magnetic cloud evolution in a two-speed solar wind. J. Geophys. Res..

[29] Cargill PJ, Chen J, Spicer DS, Zalesak ST (1996). Magnetohydrodynamic simulations of the motion of magnetic flux tubes through a magnetised plasma. J. Geophys. Res..

[30] Lugaz N, Manchester W, Gombosi T (2005). Numerical simulation of the interaction of two coronal mass ejections from Sun to Earth. The Astrophysical Journal.

[31] Xiong, M., Zheng, H., Wang, Y. & Wang, S. Magnetohydrodynamic simulation of the interaction between interplanetary strong shock and magnetic cloud and its consequent geoeffectiveness. *J. Geophys. Res*. **111**, doi:10.1029/2005JA011593 (2006).

[32] Odstrcil D, Pizzo VJ (1999). Three-dimensional propagation of coronal mass ejections (CMEs) in a structured solar wind flow: 1. CME launched within the streamer belt. J. Geophys. Res..

[33] Manchester, W. B. & Zurbuchen, T. H. Are high-latitude forward-reverse shock pairs driven by CME overexpansion? *Journal of Geophysical Research: Space Physics* **111**, n/a-n/a, doi:10.1029/2005JA011461 (2006).

[34] Riley P (2004). Fitting flux-ropes to a global MHD solution: A comparison of techniques. J. Atmos. Sol. Terr. Phys.

[35] Owens, M. J., Cargill, P. J., Pagel, C., Siscoe, G. L. & Crooker, N. U. Characteristic magnetic field and speed properties of interplanetary coronal mass ejections and their sheath regions. *J. Geophys. Res*. **110**, doi:10.1029/2004JA010814 (2005).

[36] Riley P, Crooker NU (2004). Kinematic treatment of CME evolution in the solar wind. Astrophys. J..

[37] Ruffenach A (2015). Statistical study of magnetic cloud erosion by magnetic reconnection. J. Geophys. Res..

[38] Cane HV, Richardson IG (2003). Interplanetary coronal mass ejections in the near-Earth solar wind during 1996-2002. J. Geophys. Res..

[39] Siscoe G, Odstrcil D (2008). Ways in which ICME sheaths differ from magnetosheaths. J. Geophys. Res..

[40] Eyles C (2009). The heliospheric imagers onboard the STEREO mission. Sol. Phys..

[41] Case, A., Spence, H. E., Owens, M. J., Riley, P. & Odstrcil, D. The ambient solar wind’s effect on ICME transit times. *Geophys. Res. Lett*. **35**, doi:10.1029/2008GL034493 (2008).

[42] Vrsnak B, Gopalswamy N (2002). Influence of aerodynamic drag on the motion of interplanetary ejecta. J. Geophys. Res..

